# Supplementary material for: Adolescent well-being and learning in times of COVID-19—A multi-country study of basic psychological need satisfaction, learning behavior, and the mediating roles of positive emotion and intrinsic motivation
Source: PLoS One. 2021 May 12;16(5):e0251352. doi: 10.1371/journal.pone.0251352 (PMC8115832; doi:10.1371/journal.pone.0251352)
Supplement: S1 Appendix — Each table section contains coefficients for two countries separated by the diagonal. Values in bold letters represent significant correlations at p < .05. (PDF) [file pone.0251352.s001.pdf]

S1: Appendix A

Bivariate latent correlations per country. Each table section contains coefficients for two countries separated by the diagonal. Values in bold letters represent significant correlations at p < .05.

Austria, Cyprus

	1	2	3	4	5	6	7	8	9
1. Competence	-	**.54**	**.49**	**.39**	**.55**	**.73**	.22	**-.46**	**-.23**
2. Autonomy	**.55**	-	**.33**	**.35**	**.43**	**.43**	**.34**	-.19	-.02
3. Relatedness	**.44**	**.28**	-	**.33**	**.34**	**.42**	.12	**-.38**	**-.23**
4. Positive emotion	**.69**	**.46**	**.49**	-	.17	.14	.07	-.03	-.06
5. Intrinsic learning motivation	**.66**	**.46**	**.26**	**.57**	-	**.68**	**.29**	**-.30**	**-.19**
6. Engagement	**.48**	**.32**	**.21**	**.32**	**.56**	-	.23	.14	-.02
7. Persistence	**.63**	**.35**	**.35**	**.41**	**.56**	**.67**	-	**-.54**	-.18
8. Procrastination	**-.54**	**-.27**	**-.25**	**-.34**	**-.43**	**-.38**	**-.71**	-	.16
9. Age	**-.14**	**-.06**	**-.20**	**-.19**	**-.10**	**.02**	**-.16**	**.11**	-

Note. Correlations for Austria (*N* = 19,337) are below the diagonal and correlations for Cyprus (*N* = 141) are above the diagonal.

Finland, Germany

	1	2	3	4	5	6	7	8	9
1. Competence	-	**.46**	**.38**	**.63**	**.66**	**.55**	**.68**	**-.56**	**-.12**
2. Autonomy	**.50**	-	**.20**	**.37**	**.40**	**.23**	**.35**	**-.32**	-.04
3. Relatedness	**.47**	**.27**	-	**.39**	**.13**	**.18**	**.25**	**-.18**	-.07
4. Positive emotion	**.78**	**.49**	**.57**	-	**.49**	**.26**	**.34**	**-.35**	**-.18**
5. Intrinsic learning motivation	**.71**	**.48**	**.29**	**.61**	-	**.60**	**.55**	**-.46**	-.05
6. Engagement	**.49**	**.35**	**.25**	**.40**	**.56**	-	**.68**	**-.35**	-.03
7. Persistence	**.79**	**.30**	**.36**	**.57**	**.66**	**.63**	-	**-.75**	**-.13**
8. Procrastination	**-.71**	**-.29**	**-.23**	**-.49**	**-.56**	**-.37**	**-.79**	-	**.23**
9. Age	-.03	-.01	-.03	-.06	-.02	-.04	.00	-.01	-

Note. Correlations for Finland (*N* = 614) are below the diagonal and correlations for Germany (*N* = 629) are above the diagonal.

India, North Macedonia									
	1	2	3	4	5	6	7	8	9
1. Competence	-	**.67**	**.42**	**.52**	**.71**	**.26**	**.65**	**-.39**	-.05
2. Autonomy	**.71**	-	**.29**	**.48**	**.53**	**.21**	**.42**	**-.19**	.00
3. Relatedness	**.47**	**.49**	-	**.38**	**.24**	**.09**	**.36**	**-.23**	**-.08**
4. Positive emotion	**.56**	**.50**	**.48**	-	**.50**	**.10**	**.31**	**-.17**	-.03
5. Intrinsic learning motivation	**.78**	**.64**	**.40**	**.60**	-	**.33**	**.61**	**-.29**	-.06
6. Engagement	**.60**	**.50**	**.31**	**.36**	**.59**	-	**.54**	**-.10**	**-.12**
7. Persistence	**.74**	**.62**	**.42**	**.44**	**.66**	**0.78**	-	**-.57**	**-.10**
8. Procrastination	.04	**.09**	-.01	.05	**.09**	**0.23**	.05	-	**.12**
9. Age	**-.09**	-.03	**-.10**	-.04	**-.11**	**-.09**	**-.09**	**.09**	-

Note. Correlations for India (*N* = 2,618) are below the diagonal and correlations for North Macedonia (*N* = 1,084) are above the diagonal.

Poland, USA									
	1	2	3	4	5	6	7	8	9
1. Competence	-	**.43**	**.52**	**.43**	**.48**	.05	-.05	-.04	**.14**
2. Autonomy	**.51**	-	**.30**	**.26**	**.20**	.08	.07	.05	-.03
3. Relatedness	**.43**	**.34**	-	**.54**	**.31**	**.11**	.07	-.03	-.09
4. Positive emotion	**.56**	**.28**	**.46**	-	**.28**	.07	**.12**	-.05	-.01
5. Intrinsic learning motivation	**.53**	**.51**	**.38**	**.38**	-	-.05	-.02	-.03	**.30**
6. Engagement	.11	.10	.08	.04	**.45**	-	**.31**	**-.20**	-.01
7. Persistence	**.48**	**.30**	**.35**	**.19**	**.54**	**.51**	-	**.14**	-.04
8. Procrastination	**-.49**	**-.33**	**-.31**	**-.22**	**-.48**	**-.19**	**-.73**	-	-.02
9. Age	**-.25**	**-.19**	**-.23**	**-.21**	**-.18**	.01	**-.23**	**.25**	-

Note. Correlations for Poland (*N* = 379) are below the diagonal and correlations for the USA (*N* = 503) are above the diagonal.
