# Supplementary material for: Adolescent well-being and learning in times of COVID-19—A multi-country study of basic psychological need satisfaction, learning behavior, and the mediating roles of positive emotion and intrinsic motivation
Source: PLoS One. 2021 May 12;16(5):e0251352. doi: 10.1371/journal.pone.0251352 (PMC8115832; doi:10.1371/journal.pone.0251352)
Supplement: S2 Appendix — (PDF) [file pone.0251352.s002.pdf]

S2: Appendix B

Descriptive statistics for each country of data collection

Austria								
	1	2	3	4	5	6	7	8
Number of Items	3	3	3	3	3	3	3	3
M	3.78	4.04	4.54	3.97	2.84	3.25	3.88	2.40
SD	0.92	0.97	0.75	0.94	1.15	0.93	0.82	1.11
Skewness	-0.75	-1.19	-2.12	-0.99	0.06	-0.23	-0.75	0.6
Kurtosis	0.19	0.74	4.25	0.37	-0.97	-0.59	0.03	-0.75
Range	4.00	4.00	4.00	4.00	4.00	4.00	4.00	4.00

Note. *N* = 19,337. 1. Competence, 2. Autonomy, 3. Relatedness, 4. Positive emotion, 5. Intrinsic learning motivation, 6. Engagement, 7. Persistence, 8. Procrastination.

All scales used a 5-point response format.

Cyprus								
	1	2	3	4	5	6	7	8
Number of Items	3	3	3	3	3	3	3	3
M	3.68	3.75	4.30	3.61	2.68	3.23	3.43	2.73
SD	0.98	0.83	0.73	0.96	1.04	0.98	0.88	0.90
Skewness	-0.57	-0.65	-1.26	-0.59	0.21	-0.41	-0.04	0.23
Kurtosis	-0.45	0.03	1.6	-0.32	-0.63	-0.32	-0.82	-0.93
Range	4.00	4.00	4.00	4.00	4.00	4.00	4.00	4.00

Note. *N* = 141. 1. Competence, 2. Autonomy, 3. Relatedness, 4. Positive emotion, 5. Intrinsic learning motivation, 6. Engagement, 7. Persistence, 8. Procrastination.

All scales used a 5-point response format.

Finland								
	1	2	3	4	5	6	7	8
Number of Items	3	3	3	3	3	3	3	3
M	3.34	3.03	4.03	3.43	2.70	3.01	3.27	3.20
SD	1.01	0.97	0.79	0.85	0.92	0.82	0.86	1.12
Skewness	-0.51	-0.13	-1.12	-0.29	0.05	0.00	-0.314	-0.12
Kurtosis	-0.59	-0.67	1.52	-0.35	-0.63	-0.26	-0.39	-0.97
Range	4.00	4.00	4.00	4.00	4.00	4.00	4.00	4.00

Note. *N* = 614. 1. Competence, 2. Autonomy, 3. Relatedness, 4. Positive emotion, 5. Intrinsic learning motivation, 6. Engagement, 7. Persistence, 8. Procrastination.

All scales used a 5-point response format.

Germany								
	1	2	3	4	5	6	7	8
Number of Items	3	3	3	3	3	3	3	3
M	3.43	3.74	4.39	3.66	2.45	2.98	3.58	2.83
SD	0.94	1.01	0.80	0.95	1.03	0.94	0.85	1.15
Skewness	-0.40	-0.77	-1.63	-0.60	0.40	0.11	-0.40	0.19
Kurtosis	-0.33	-0.16	2.3	-0.36	-0.65	-0.53	-0.45	-1.10
Range	4.00	4.00	4.00	4.00	4.00	4.00	4.00	4.00

Note. *N* = 629. 1. Competence, 2. Autonomy, 3. Relatedness, 4. Positive emotion, 5. Intrinsic learning motivation, 6. Engagement, 7. Persistence, 8. Procrastination.

All scales used a 5-point response format.

India								
	1	2	3	4	5	6	7	8
Number of Items	3	3	3	3	3	3	3	3
M	3.63	3.86	4.08	3.73	3.35	3.56	3.76	3.46
SD	0.90	0.79	0.77	0.74	1.01	0.74	0.80	0.75
Skewness	-0.69	-0.95	-1.12	-0.68	-0.43	-0.49	-0.69	-0.41
Kurtosis	0.04	1.07	1.33	0.30	-0.57	-0.15	0.36	-0.37
Range	4.00	4.00	4.00	4.00	4.00	4.00	4.00	4.00

Note. *N* = 2,618. 1. Competence, 2. Autonomy, 3. Relatedness, 4. Positive emotion, 5. Intrinsic learning motivation, 6. Engagement, 7. Persistence, 8. Procrastination.

All scales used a 5-point response format.

North Macedonia								
	1	2	3	4	5	6	7	8
Number of Items	3	3	3	3	3	3	3	3
M	3.72	3.73	4.41	3.86	3.06	3.32	3.84	2.61
SD	0.89	0.91	0.79	0.87	1.10	0.88	0.81	1.04
Skewness	-0.73	-0.78	-1.83	-0.87	-0.06	-0.19	-0.71	0.45
Kurtosis	0.18	0.21	3.66	0.51	-0.84	-0.64	0.32	-0.77
Range	4.00	4.00	4.00	4.00	4.00	4.00	4.00	4.00

Note. *N* = 1,084. 1. Competence, 2. Autonomy, 3. Relatedness, 4. Positive emotion, 5. Intrinsic learning motivation, 6. Engagement, 7. Persistence, 8. Procrastination.

All scales used a 5-point response format.

Poland								
	1	2	3	4	5	6	7	8
Number of Items	2*	3	3	3	3	3	3	3
M	3.56	3.20	4.12	3.52	2.47	3.13	3.43	3.02
SD	1.03	1.05	0.92	0.94	1.02	0.89	0.81	1.02
Skewness	-0.50	-0.20	-1.28	-0.51	0.41	-0.05	-0.31	0.13
Kurtosis	-0.22	-0.82	1.29	-0.36	-0.52	-0.42	-0.27	-0.93
Range	4.00	4.00	4.00	4.00	4.00	4.00	4.00	4.00

Note. *N* = 379. 1. Competence, 2. Autonomy, 3. Relatedness, 4. Positive emotion, 5. Intrinsic learning motivation, 6. Engagement, 7. Persistence, 8. Procrastination.

All scales used a 5-point response format.

* Due to technical issues, participants in Poland were only presented two items of the competence scale.

USA								
	1	2	3	4	5	6	7	8
Number of Items	3	3	3	3	3	3	3	3
M	3.53	3.63	3.62	3.24	3.76	3.32	3.25	3.13
SD	1.40	1.09	1.29	1.31	1.25	1.15	0.98	1.02
Skewness	-0.53	-0.64	-0.59	-0.15	-0.73	-0.40	-0.10	0.05
Kurtosis	-1.38	-0.50	-1.28	-1.48	-0.89	-0.90	-0.71	-0.87
Range	4.00	4.00	4.00	4.00	4.00	4.00	4.00	4.00

Note. *N* = 503. 1. Competence, 2. Autonomy, 3. Relatedness, 4. Positive emotion, 5. Intrinsic learning motivation, 6. Engagement, 7. Persistence, 8. Procrastination.

All scales used a 5-point response format.
