# Supplementary material for: Adolescent well-being and learning in times of COVID-19—A multi-country study of basic psychological need satisfaction, learning behavior, and the mediating roles of positive emotion and intrinsic motivation
Source: PLoS One. 2021 May 12;16(5):e0251352. doi: 10.1371/journal.pone.0251352 (PMC8115832; doi:10.1371/journal.pone.0251352)
Supplement: S3 Appendix — (PDF) [file pone.0251352.s003.pdf]

S3: Appendix C

95% Bias-corrected bootstrap confidence intervals for direct and indirect effects of the multi-group mediation model for each country of data collection

	Austria		Cyprus		Finland		Germany		India		North Macedonia		Poland*		USA	
	Lower 2.5%	Upper 2.5%.	Lower 2.5%	Upper 2.5%.	Lower 2.5%	Upper 2.5%.	Lower 2.5%	Upper 2.5%.	Lower 2.5%	Upper 2.5%.	Lower 2.5%	Upper 2.5%.	Lower 2.5%	Upper 2.5%.	Lower 2.5%	Upper 2.5%.
**Direct Effects**																
Positive Emotion → Engagement	-0.10	-0.07	-0.19	0.12	-0.22	0.05	-0.22	-0.06	-0.10	0.03	-0.10	0.04	-0.16	0.03	-0.06	0.11
Positive Emotion → Persistence	-0.12	-0.09	-0.24	0.02	-0.28	0.04	-0.23	-0.06	-0.12	0.03	-0.15	-0.01	-0.21	-0.04	-0.01	0.20
Positive Emotion → Procrastination	0.09	0.15	-0.15	0.24	-0.06	0.36	-0.07	0.23	-0.04	0.15	-0.04	0.18	0.02	0.29	-0.16	0.09
Learning Motivation →Engagement	0.24	0.26	-0.09	0.30	0.19	0.36	0.20	0.37	0.08	0.18	0.07	0.21	0.22	0.50	-0.16	0.01
Learning Motivation → Persistence	0.16	0.19	0.08	0.42	0.07	0.29	0.05	0.25	0.06	0.18	0.12	0.26	0.17	0.39	-0.11	0.10
Learning Motivation → Procrastination	-0.19	-0.14	-0.20	0.25	-0.31	0.01	-0.36	-0.05	0.01	0.16	-0.17	0.06	-0.55	-0.17	-0.10	0.15
Competence → Engagement	0.16	0.20	-0.23	0.33	-0.01	0.25	0.16	0.38	0.11	0.27	-0.07	0.17	-0.19	0.07	-0.09	0.11
Competence → Persistence	0.37	0.42	0.18	0.73	0.44	0.75	0.35	0.64	0.32	0.51	0.23	0.48	0.09	0.39	-0.25	-0.02
Competence → Procrastination	-0.72	-0.64	-0.68	-0.08	-1.18	-0.74	-0.81	-0.37	-0.19	0.02	-0.72	-0.33	-0.64	-0.24	-0.20	0.09
Competence → Positive Emotion	0.59	0.64	-0.13	0.57	0.45	0.63	0.47	0.73	0.23	0.38	0.19	0.46	0.36	0.73	0.07	0.36
Competence → Learning Motivation	0.79	0.84	0.20	0.81	0.55	0.74	0.68	0.92	0.73	0.94	0.74	1.04	0.19	0.47	0.21	0.47
Autonomy → Engagement	0.00	0.03	-0.02	0.51	-0.03	0.12	-0.12	0.03	0.01	0.15	-0.06	0.11	-0.20	0.00	-0.06	0.14
Autonomy → Persistence	-0.04	0.00	-0.25	0.25	-0.22	-0.04	-0.05	0.12	0.05	0.22	-0.10	0.09	-0.19	0.05	-0.02	0.22
Autonomy → Procrastination	0.03	0.09	-0.17	0.51	0.00	0.26	-0.23	0.04	0.03	0.23	0.02	0.30	-0.16	0.21	-0.05	0.24
Autonomy → Positive Emotion	0.10	0.14	-0.08	0.71	0.03	0.20	-0.01	0.22	0.01	0.20	0.14	0.38	-0.22	0.11	-0.06	0.27
Autonomy → Learning Motivation	0.17	0.22	-0.11	0.59	0.10	0.29	0.02	0.24	0.04	0.26	0.01	0.28	0.18	0.48	-0.14	0.13
Relatedness → Engagement	0.03	0.06	-0.25	0.26	-0.04	0.14	-0.01	0.14	-0.03	0.06	-0.06	0.06	-0.11	0.08	-0.04	0.17
Relatedness → Persistence	0.09	0.13	-0.17	0.30	-0.07	0.14	-0.05	0.16	0.00	0.10	0.07	0.21	0.00	0.22	0.07	0.18
Relatedness → Procrastination	-0.10	-0.04	-0.54	0.10	0.00	0.27	-0.14	0.17	-0.13	0.01	-0.22	-0.01	-0.26	0.05	-0.16	0.13
Relatedness → Positive Emotion	0.26	0.31	-0.16	0.59	0.17	0.36	0.10	0.32	0.18	0.30	0.15	0.34	0.15	0.46	0.31	0.62
Relatedness → Learning Motivation	-0.09	-0.05	-0.18	0.37	-0.20	0.02	-0.30	-0.09	-0.07	0.06	-0.18	-0.01	0.01	0.25	0.01	0.25

**Indirect Effects**																
Competence → Positive Emotion																
Engagement	-0.06	-0.04	-0.06	0.02	-0.12	0.02	-0.14	-0.04	-0.03	0.01	-0.04	0.01	-0.09	0.01	-0.01	0.03
Persistence	-0.08	-0.05	-0.12	0.01	-0.16	0.02	-0.15	-0.04	-0.04	0.01	-0.06	0.00	-0.13	-0.02	0.00	0.06
Procrastination	0.06	0.09	-0.03	0.12	-0.03	0.21	-0.04	0.14	-0.01	0.05	-0.01	0.07	0.01	0.18	-0.04	0.02
Competence → Learning Motivation																
Engagement	0.19	0.22	-0.03	0.20	0.12	0.25	0.16	0.31	0.06	0.16	0.07	0.19	0.06	0.20	-0.06	0.00
Persistence	0.13	0.15	0.03	0.28	0.05	0.19	0.04	0.20	0.05	0.15	0.11	0.24	0.05	0.15	-0.04	0.03
Procrastination	-0.15	-0.12	-0.09	0.14	-0.20	0.01	-0.28	-0.04	0.01	0.14	-0.15	0.05	-0.21	-0.05	-0.04	0.05
Autonomy → Positive Emotion																
Engagement	-0.01	-0.01	-0.12	0.02	-0.04	0.00	-0.04	0.00	-0.02	0.00	-0.03	0.01	0.00	0.03	-0.01	0.02
Persistence	-0.02	-0.01	-0.13	0.01	-0.05	0.00	-0.04	0.00	-0.02	0.00	-0.05	0.00	-0.01	0.04	0.00	0.04
Procrastination	0.01	0.02	-0.03	0.15	0.00	0.06	-0.01	0.04	0.00	0.02	-0.01	0.05	-0.05	0.01	-0.04	0.01
Autonomy → Learning Motivation																
Engagement	0.04	0.06	-0.02	0.14	0.03	0.09	0.01	0.08	0.01	0.04	0.00	0.05	0.06	0.21	-0.01	0.02
Persistence	0.03	0.04	-0.01	0.20	0.01	0.07	0.00	0.05	0.00	0.04	0.00	0.06	0.04	0.16	-0.01	0.01
Procrastination	-0.04	-0.03	-0.06	0.10	-0.07	0.00	-0.07	0.00	0.00	0.03	-0.04	0.01	-0.22	-0.05	-0.01	0.01
Relatedness → Positive Emotion																
Engagement	-0.03	-0.02	-0.09	0.02	-0.06	0.01	-0.06	-0.01	-0.03	0.01	-0.03	0.01	-0.06	0.01	-0.03	0.05
Persistence	-0.04	-0.03	-0.11	0.01	-0.08	0.01	-0.06	-0.01	-0.03	0.01	-0.04	0.00	-0.08	-0.01	0.00	0.10
Procrastination	0.03	0.04	-0.03	0.12	-0.01	0.10	-0.01	0.05	-0.01	0.04	-0.01	0.05	0.01	0.11	-0.08	0.04
Relatedness → Learning Motivation																
Engagement	-0.02	-0.01	-0.02	0.09	-0.06	0.00	-0.10	-0.03	-0.01	0.01	-0.03	0.00	0.01	0.11	-0.03	0.00
Persistence	-0.02	-0.01	-0.04	0.11	-0.05	0.00	-0.06	-0.01	-0.01	0.01	-0.04	0.00	0.01	0.08	-0.02	0.01
Procrastination	0.01	0.02	-0.03	0.06	0.00	0.05	0.01	0.09	-0.01	0.01	0.00	0.02	-0.11	-0.01	-0.01	0.03

*As participants in Poland, due to technical issues, were only presented two items of the competence scale, the model for Poland was not analyzed within the multi-group model but separately.
