# Supplementary material for: Adolescent well-being and learning in times of COVID-19—A multi-country study of basic psychological need satisfaction, learning behavior, and the mediating roles of positive emotion and intrinsic motivation
Source: PLoS One. 2021 May 12;16(5):e0251352. doi: 10.1371/journal.pone.0251352 (PMC8115832; doi:10.1371/journal.pone.0251352)
Supplement: Attachment — Submitted filename: Response to Reviewers.docx [file pone.0251352.s004.docx]

**Protocol of changes and improvements in accordance with the reviewers’ recommendations**

**To the Editor:**

We wish to thank the editor and the reviewers for their positive assessment of our manuscript and for the feedback, which helped us to further improve our work. This is much appreciated. Below, we address the reviewers’ comments. For each of the comments, we present our answers in detail and point out the corresponding changes in the revised manuscript (parts that have been deleted are crossed out, parts that have been added are underlined). Within the main manuscript, we have accordingly highlighted the changes by using track changes mode. From our perspective, the quality of the paper has been significantly improved. We thank the editor and the reviewers for their time and attention devoted to this manuscript and hope to obtain a favorable review.

**To Reviewer 1:**

***Comments to the Author***

I have read the paper on adolescent well-being and learning in times of COVID-19 with great interest. I found the manuscript to be very well developed and to address a highly important and relevant topic. Furthermore, I also found the manuscript to be very well written, clearly argued for, and well discussed. Notable strengths of the manuscript entail its large data-set, the rigorous testing of central assumptions (such as measurement invariance), and the detailed description of the findings. I believe that the manuscript will be very well received by readers of PLOS ONE and congratulate the authors to an important contribution to the literature. I only have a very few points that the authors may want to consider when revising their manuscript.

We thank the reviewer very much for the very kind feedback and for the suggestions for improvement, which we address in detail below.

**[R1 – Comment #1]**

Page 5: Advantages of autonomy-supportive learning environments: These also require the students to have the necessary SRL skills (which, they frequently, do not have). Therefore, learning environments should critically consider such aspects, e.g. by prompting students to employ adequate learning strategies. The authors may want to consider this point by relativizing their argument.

Thank you for pointing this out. We have relativized the argumentation accordingly.

See page 5:

~~When implemented accordingly, d~~ Distance education may provide learners with manifold opportunities to practice, test, and expand their knowledge at their own pace [37]. This allows to optimally challenge students and to achieve a high degree of individualization of their learning processes [38, 39]. However, individualized learning also requires the learner to be able to handle flexibility and not all learners are able to cope with distance learning equally well [40]. Students in distance learning environments should therefore be encouraged to employ adequate learning strategies with respect to self-regulation [41]. When implemented successfully, individualized, autonomy-supportive learning environments have consistently been shown to create optimal conditions for learners to experience competence [17].

**[R1 – Comment #2]**

Page 6: “To the best of our knowledge …” + next sentence: These two sentences seem to contradict each other. Please rephrase to clarify this.

The two sentences have been changed in order to point out that there is now, in contrast to past epidemics, a growing body of research on this topic. We hope that the contradiction of these two sentences is now resolved by adding the words “now” and “past”.

See page 6 in the manuscript:

To the best of our knowledge, no prior research has been conducted to explore SDT in the context of past ~~an~~ epidemics (e.g., SARS). However, there now is a growing body of studies conducted during the COVID-19 pandemic across different cultures [54, 58, 59], pointing to the universal applicability of SDT even in exceptional situations like a pandemic.

**[R1 – Comment #3]**

Generally, I recommend to write about “competence satisfaction” instead of solely “competence” throughout the manuscript (also regarding autonomy and relatedness of course) in order to be clear in writing and avoid misunderstandings.

We thank the reviewer for this important note, as this might cause misinterpretations. The respective phrases were changed by explicitly referring to basic psychological need satisfaction, experienced or perceived competence, as well as perceived or experienced autonomy and perceived or experienced relatedness throughout the manuscript.

**[R1 – Comment #4]**

I was wondering how the students were recruited, particularly with regard to them possibly being from different classrooms. If they are from different classrooms, it might be helpful to provide information on the ICCs of the assessed constructs to make sure that there are no substantial amounts of shared variance that could cause problems with model estimation.

For data collection, we have sent the link to the online questionnaire to schools and various stakeholders, along with the request to forward it to students. However, in order to ensure complete anonymity according to the European General Data Protection Regulation, we have not asked students about where they go to school and what class they attend within the questionnaire. Thus, the data does not allow to control for nesting of the data (classes, schools). However, we had no research questions on the school or class level. We now address this issue in the limitations section.

See page 30 in the manuscript:

It would also be profitable to explicitly consider the multilevel structure of the data (students in classes, classes in schools) to identify possible context effects. This could not be controlled for in this study, due to the anonymization of the data.

**[R1 – Comment #5]**

The CFI and RMSEA cut-offs by Chen seem very adequate, especially as Chi²-differnece tests would be biased by the large sample sizes. The cut-offs could be used in an even more detailed manner. Chen recommends partly different cut-offs for the different steps of measurement invariance testing.

We followed the recommendations of Chen (2007) for a sample size with total N > 300. *“For testing loading invariance, a change of ≥ -.010 in CFI, supplemented by a change of ≥.015 in RMSEA or a change of* ≥ *.030 in SRMR would indicate noninvariance; for testing intercept or residual invariance, a change of ≥ -.010 in CFI, supplemented by a change of ≥ .015 in RMSEA or a change of ≥ .010 in SRMR would indicate noninvariance. In other words, similar values are suggested for CFI and RMSEA across all three levels of invariance tests, but different values are proposed for SRMR, as SRMR is more sensitive to noninvariance in loadings than to noninvariance in intercepts or residual variances.”* (p. 501)

As we only used CFI and RMSEA, the cut-offs are the same for all steps of MI testing.

**[R1 – Comment #6]**

Note of Table 4: Were the items or the answers from Poland not included? Please clarify what “they” refers to accordingly.

Thank you very much for bringing this to our attention. We have clarified the statement accordingly.

See Note of Table 4:

As participants in Poland, due to technical issues, were only presented two items of the competence scale, ~~they~~ Polish data were not included in the measurement invariance analysis for the predictors.

**[R1- Comment #7]**

Differences between different countries in model path estimates: As some of the countries have much smaller sample size than others, it is not surprising that some parameter estimates are not significant, despite being descriptively in the same order of magnitude as parameter estimates from larger countries (e.g. second parameter estimate for Austria and Cyprus). Power analyses could consequently be included to make a stronger points that this does not imply that there are meaningful differences between the different countries.

We agree with the reviewer that based on the differences in sample sizes, it is unsurprising that smaller effects were detected in countries with larger samples, rather than in countries with smaller samples. Due to the sudden occurrence of the distance learning situation, the preparation time for developing the present study’s research design was very limited. Given these circumstances, we relied on ad hoc samples, rather than recruiting sample sizes based on prospective power analyses. In line with the reviewer’s remark, we have been considering different ways to deal with the limitation of different sample sizes. Our review of current literature showed that post hoc power analyses are not recommended for that purpose (Cumming & Calin-Jageman, 2017; Kraemer, 2013; Zhang et al., 2019). Accordingly, it is suggested not to conduct power analyses after a study has been conducted and a statistical decision has been made. This is (also) why we report the confidence intervals of the effects in this study. They not only provide information on which results differ statistically significantly from one another. Their size also accounts for the statistical power of the respective samples.

Cumming, G., & Calin-Jageman, R. (2017). *Introduction to the New Statistics. Estimation, Open Science, and Beyond*. Routledge.

Kraemer, H. C. (2013). Statistical Power: Issues and Proper Applications. In J. S. Comer & P. C. Kendall (Eds.), *The Oxford Handbook of Research Strategies for Clinical Psychology* (pp. 213–226). Oxford University Press.

Zhang, Y., Hedo, R., Rivera, A., Rull, R., Richardson, S., & Tu, X. M. (2019). Post hoc power analysis: is it an informative and meaningful analysis? *General Psychiatry*, *32*(4), e100069. https://doi.org/10.1136/gpsych-2019-100069

**R1 – Comment #8**]

I was surprised that the differences in parameter estimates were based on the confidence intervals. The most straightforward approach to me would have been to extend the logic of measurement invariance testing and restrict the parameters to be equal across the different groups. If full invariance is not attested, restrictions can be loosened until partial invariance is reached. The authors may wish to consider this or to provide a stronger rationale for the approach that they used (possible, due to the large amount of countries, the first approach that I suggested by not be so sensible after all).

Thank you for that comment. It was also our initial approach to restrict the slopes to be equal across countries. However, this lead to a substanstial decrease in model fit as all slopes were different from another. We then attempted to freely estimate the slopes of different combinations of countries. However, due to the large amount of countries, this did not turn out to be an efficient approach. As a pragmatic approach to compare effect by effect we finally decided for using the confidence intervals of the estimates. This made it possible to gain a very clear overview of which effects differ from one another, taking into account the different statistical power of the respective samples. We have now also specified this in the methods section.

See page 13 in the manuscript:

In a final step, we ~~to~~ explored common findings and discrepancies across countries and examined whether countries differed from one another in terms of the identified effect sizes. Due to the large number of countries, we aimed for an approach that allows to gain a clear overview of which effects differ from one another, taking into account the different statistical power of the respective samples. We therefore used the bootstrapping confidence intervals of the direct and indirect effects ~~were further used~~ to examine whether countries differed from one another in terms of the identified effect sizes. Accordingly, if intervals overlap, i.e., enclose a common range of values, they indicate that the difference between countries is not statistically significant. If there is no overlap, the difference is statistically significant. ~~overlapping confidence intervals indicate that effects do not significantly differ from one another, and vice versa.~~

**To Reviewer 2:**

***Comments to the Author***

This is an interesting and timely study which examines adolescents’ well-being during Covid-19 pandemic across eight countries using a large sample size which further strengthens findings and generalizability of the findings. Here are my comments and suggestions for the authors that I hope they find helpful:

We would like to thank the reviewer for the feedback and suggestions for improvement, which we address in detail below.

**[R2 – Comment #1]**

Except for Cyprus which has an age range of 12-17, other countries have the maximum age of for example, 20 or 21. I’m not sure whether a person whose age is 21 is attending a secondary school. This might be true, but at least some clarification might be necessary here to clarify the different contexts.

Thank you for this comment. Indeed, it is possible for students to attend secondary school even at the age of 21. In Austria, this is for example the case when students attend a so-called “higher vocational school” that lasts 5 years instead of the usual 4 years of upper secondary education. Additionally, students in Austria may repeat school grades up to 2 times. Repeating school grades several times, e.g. due to failing exams, is also possible in other countries of data collection. Accordingly, in some cases, secondary students may be 21 years old. However, this is true for only a very small proportion of participants in our study. We have addressed the reviewer's comment in the description of the sample.

See page 8 in the manuscript:

The overall sample comprised 25,305 ~~secondary school~~ students (37.9% males, 61.5% females, 0.6% diverse). Their mean age was 14.86 years (*SD* = 2.43, *Mdn* = 15.00, *Range* = 10 - 21). All students reported attending secondary schools. Their age range reflects the fact that in many countries of data collection, students older than 18 may still attend secondary education e.g., if the school form integrates vocational training [62] or due to grade repetition.

**[R2 – Comment #2**]

Did authors control for the age? As it can be seen from Table 1, the age range for some countries is between 10-19 or 10-21. It seems this age range includes more than adolescents and it can affect the analyses.

We agree with the reviewer and reran the mediation analyses, controlling for the effect of age for all paths examined. The entire results section as well as Table 5 and Appendix C, accounting for the confidence intervals, have been revised (see results section and Appendix C). There were minimal changes in the estimates, mostly only with respect to the second decimal place. In total, 6 small effects became statistically significant after controlling for age, and 3 small effecs were not statistically significant anymore. We now also report the correlations of the investigated constructs with age in Appendix A and refer to controlling for age in the hypotheses section:

See page 8:

In line with previous studies that indicate associations of age with intrinsic learning motivation [60] and positive emotion [61], we treated participants’ age as control variables.

Due to slightly different results in terms of statistical significance, the following small changes were implemented in the discussion section:

See page 25:

While ~~a~~ small, positive effects were ~~was~~ identified in Poland and the USA, there were small, negative effects of experienced relatedness on intrinsic learning motivation in Austria, Germany and North Macedonia.

See page 26:

In some countries, the results contradicted the hypotheses: A small, but positive association between intrinsic learning motivation and procrastination emerged in India, negative effects of positive emotion on engagement arose in Austria and Germany, and negative effects of positive emotion on persistence were shown in Austria, Germany, North Macedonia and Poland.

See page 27:

Also in line with these possible explanations, there were ~~was a~~ positive, small effects of positive emotion on procrastination in Austria and India. ~~, which, however, was not significant in any other country.~~

See page 28:

In contrast, the highest proportions of explained variance emerged for Finland, followed by Germany, Austria, India, Poland, North Macedonia, and Cyprus ~~and North Macedonia~~.

**[R2 – Comment #3]**

Authors stated that they used bootstrapping for testing significance. As far as I now, MLR cannot produce bootstrapping confidence intervals. Can authors explain how they obtained bootstrapped confidence intervals with MLR? Did they run the models again?

You are right, applying both MLR and bootstrapping in the same analyses is not possible. We therefore applied MLR only in the CFAs, but not for the mediation analysis. We are sorry for having created confusion with this misleading description of our analyses. We now explicitly state that the MLR estimator was used for the preliminary analyses, whereas the ML estimator was used for the main analyses.

See page 12 in the manuscript:

Preliminary analyses to ensure internal consistency (CR; [68]) and construct validity (CFAs) were conducted applying robust maximum likelihood estimation (MLR).

See page 13 in the manuscript:

For testing significance of direct and indirect effects within the mediation models, we used bias-corrected bootstrapping confidence intervals based on 10,000 bootstrap draws and applied maximum likelihood estimation (ML).

**[R2 – Comment # 4]**

It might be helpful to include ∆CFI and ∆RMSEA in Table 4.

Following the reviwer’s suggestion, we have included ∆CFI and ∆RMSEA in Table 4.

**[R2 – Comment #5]**

Concerning Table 5, it seems authors have made significant relations bold, but this has not been mentioned in the note of Table 5.

We thank the reviewer for drawing our attention to the missing note. The note has been changed accordingly by adding “Statistically significant results with α < .05 are in boldface” (see Table 5).

**[R2 – Comment #6]**

Regarding H7, authors state that overlapping confidence intervals did not differ from one another. Can authors clarify what does exactly “overlapping confidence intervals” mean?

We have now clarified the term in the methods section.

See page 14 in the manuscript:

Accordingly, if intervals overlap, i.e., enclose a common range of values, they indicate that the difference between countries is not statistically significant. If there is no overlap, the difference is statistically significant. ~~overlapping confidence intervals indicate that effects do not significantly differ from one another, and vice versa.~~

**[R2 – Comment #7**]

While the text is generally well-written, there are some linguistic issues with regard to the text which need to be revised. Here are some examples:
“data were collected in altogether eight countries in Europe, Asia and North America” -> I think this sentence needs to be revised. My suggestion: “data were collected in altogether FROM eight countries in Europe, Asia and North America”.

Thank you for pointing this out. We have revised the manuscript accordingly.

See Abstract:

Data were collected via online questionnaires in altogether eight countries ~~in~~ from Europe, Asia, and North America (N = 25,305) and comparable results across countries were expected.

See page 3:

To take a broad perspective and to derive common conclusions applicable across different cultural contexts, data were collected in altogether eight countries ~~in~~ from Europe, Asia and North America.

See page 8:

To further account for a possible universality of the findings, data were collected in altogether eight countries ~~in~~ from Europe, Asia and North America, namely Austria, Cyprus, Finland, Germany, India, North Macedonia, Poland and the USA.

See page 25:

Having collected data in altogether eight countries ~~in~~ from Europe, Asia, and North America, we expected to obtain comparable results across the countries of data collection.

**[R2 – Comment #8]**

P.10: “Before being forwarded to the items, …” -> I can’t understand this phrase. Do authors mean “Before answering the items,…”?

Yes, this phrase should express that participants were informed about the study’s goals before answering the items. We changed this sentence in accordance with the reviewer’s suggestion.

See page 9 in the manuscript:

Before ~~being forwarded to~~ answering the items, participants were informed about the study’s goals, expected duration of filling in the questionnaire, inclusion criteria for participation (i.e., attending secondary school in the respective country), and the complete anonymity of their data.

**[R2 – Comment #9]**

“The statistically significant effects that were identified in seven, respectively all eight countries, were predominantly large.” -> this sentence needs to be revised.

Thank you for that remark. The sentence has been revised.

See page 25 in the manuscript:

With regard to hypothesis set 1 (i.e., direct effects of basic need satisfaction on the mediators), the effects of experienced competence on positive emotion and intrinsic learning motivation proved empirically most robust across the countries of data collection. ~~The s~~Statistically significant effects ~~that~~ were identified in seven, respectively all eight countries. ~~and~~ ~~t~~These effects were predominantly large.

**[R2 – Comment #10]**

 “Unfavorable effects of autonomy have so far primarily been drawn attention to in the context of occupational psychology” -> this sentence needs to be revised.

Thank you for the feedback. We revised the sentence as follows:

See page 26 in the manuscript:

~~Unfavorable effects of autonomy have so far primarily been drawn attention to in the context of occupational psychology~~

So far, unfavorable effects of autonomy have primarily been investigated in the context of occupational psychology [82], but should also be considered in the context of (distance) education.

**Further Changes**

Since the current study was carried out as part of a larger research project, related works have been published since the first submission of the present manuscript. We have therefore added this information in the methods section:

See page 9 in the manuscript:

It was carried out as part of a larger research project focused on “Learning under COVID-19 circumstances” [63-66]

**Journal Requirements**

Please ensure that your manuscript meets PLOS ONE's style requirements, including those for file naming. The PLOS ONE style templates can be found at https://journals.plos.org/plosone/s/file?id=wjVg/PLOSOne_formatting_sample_main_body.pdf and https://journals.plos.org/plosone/s/file?id=ba62/PLOSOne_formatting_sample_title_authors_affiliations.pdf

We have reviewed our materials with this in mind and hope that we have not overlooked anything.

You indicated that you had ethical approval for your study. In your Methods section, please ensure you have also stated whether you obtained consent from parents or guardians of the minors included in the study or whether the research ethics committee or IRB specifically waived the need for their consent.

Thank you for that remark. We have now clarified this issue.

See page 9 in the manuscript:

Due to the special circumstances of data collection (i.e., nationwide lockdowns) and the fact that the questionnaire did not deal with any sensitive issues, the Ministry of Education carefully considered the content of the study, its possible benefits, and authorized the study to be conducted without the explicit consent of participants' legal guardians.

In your Data Availability statement, you have not specified where the minimal data set underlying the results described in your manuscript can be found. PLOS defines a study's minimal data set as the underlying data used to reach the conclusions drawn in the manuscript and any additional data required to replicate the reported study findings in their entirety. All PLOS journals require that the minimal data set be made fully available. For more information about our data policy, please see <http://journals.plos.org/plosone/s/data-availability>. Upon re-submitting your revised manuscript, please upload your study’s minimal underlying data set as either Supporting Information files or to a stable, public repository and include the relevant URLs, DOIs, or accession numbers within your revised cover letter. For a list of acceptable repositories, please see http://journals.plos.org/plosone/s/data-availability#loc-recommended-repositories. Any potentially identifying patient information must be fully anonymized.

We have deposited our data as well as the codebook in a public repository. We have already provided a private, view-only link in the first submission. We have now set the settings to public to cite the dataset and codebook with a doi. This has also been remarked within the updated data availability statement as well as in the cover letter.
